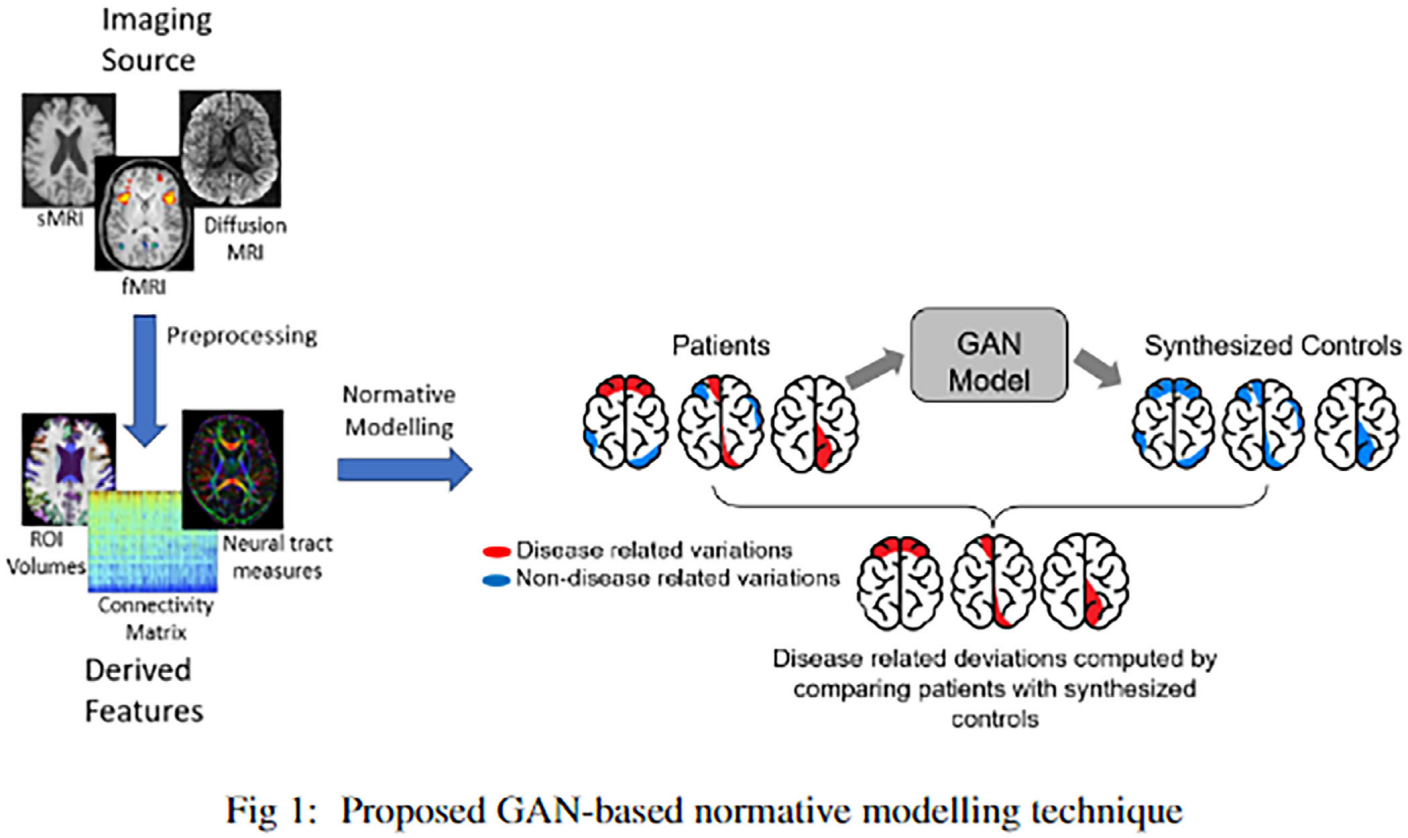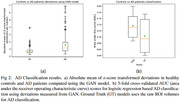# Parsing disease heterogeneity using normative modelling and Generative Adversarial Networks (GANs)

**DOI:** 10.1002/alz.087833

**Published:** 2025-01-09

**Authors:** Sai Spandana Chintapalli, Sindhuja Tirumalai Govindarajan, Haochang Shou, Hao Huang, Christos Davatzikos

**Affiliations:** ^1^ University of Pennsylvania, Philadelphia, PA USA; ^2^ Artificial Intelligence in Biomedical Imaging Laboratory (AIBIL), Center for and Data Science for Integrated Diagnostics (AI2D), Perelman School of Medicine, University of Pennsylvania, Philadelphia, PA USA; ^3^ Centre for Biomedical Image Computing and Analytics, University of Pennsylvania, Philadelphia, PA USA; ^4^ Children’s Hospital of Philadelphia, Philadelphia, PA USA; ^5^ Artificial Intelligence in Biomedical Imaging Laboratory (AIBIL), Perelman School of Medicine, University of Pennsylvania, Philadelphia, PA USA

## Abstract

**Background:**

Structural and functional heterogeneity in the brains of patients with Alzheimer's disease (AD) leads to diagnostic and prognostic uncertainty and confounds clinical treatment planning. Normative modelling, where individual‐level deviations in brain measures from a reference sample are computed to infer personalized effects of disease, allows parsing of disease heterogeneity. In this study, GAN based normative modelling technique quantifies individual level neuroanatomical abnormality thereby facilitating measurement of personalized disease related effects in AD patients.

**Method:**

We adapt the pix2pix GAN to translate a subject with disease to a corresponding subject without disease. We train this model using a dataset comprising of healthy controls and synthetically simulated patients. Healthy controls (n=6000) are selected from the ISTAGING consortium. Our neuroanatomical brain measures are the 10 region of interest (ROI) volumes (covering left and right frontal, parietal, occipital, temporal lobes as well as deep brain structures) computed using a multi‐atlas segmentation technique. To simulate patients, for each healthy control we introduce 10‐30% atrophy or expansion in a random combination of ROIs while preserving clinical covariate effects. The model learns to synthesize patient‐specific controls by removing disease‐related variations from patient’s brain measures (Figure 1). Deviation of the patient from the synthesized disease‐free control acts as an image‐based biomarker that is sensitive to disease effects and severity. For performance assessment, we select 200 controls (CN) and 200 AD participants (PT) from the OASIS dataset and compute their deviations across the 10 ROI volumes using the model pretrained on the ISTAGING dataset. Logistic regression is used to assess the overall discriminative power of the derived deviations in AD classification.

**Result:**

Larger deviations in PT compared to CN suggest disease related abnormality in brain measures (Figure 2.a). Additionally, GAN’s deviations provide a considerable gain over raw ROI volumes in AD classification as quantified using the 5‐fold AUC scores (Figure 2.b).

**Conclusion:**

GAN‐based normative modelling technique introduced here is a useful tool to parse heterogeneity in brain measures at an individual level. We see that self‐supervised training of the model using pseudo‐synthetically simulated patient data that is agnostic to disease patterns can help detect real disease related effects.